# Metabolic and Anti-Inflammatory Effects of Berberine—Rationale for Its Therapeutic Potential in Polycystic Ovary Syndrome

**DOI:** 10.3390/ijms27167287

**Published:** 2026-08-15

**Authors:** Dariusz Szukiewicz

**Affiliations:** Department of Biophysics, Physiology & Pathophysiology, Faculty of Health Sciences, Medical University of Warsaw, 02-004 Warsaw, Poland; dariusz.szukiewicz@wum.edu.pl

**Keywords:** berberine, bioactive isoquinoline alkaloid, antiandrogenic properties, androgen receptor suppression, polycystic ovary syndrome, natural metabolic modulator, anti-inflammatory compound, natural antidiabetic compound, natural antioxidant

## Abstract

Polycystic ovary syndrome (PCOS) is a common hormonal disorder in women of reproductive age and is characterized by ovarian hyperandrogenism and irregular ovulation. This often leads to infertility. Beyond reproduction, PCOS causes widespread metabolic issues such as insulin resistance (IR), increasing long-term risks for diabetes, obesity, and heart disease. In a vicious cycle, IR acts as a core driver of both clinical symptoms and associated obesity, whereas compensatory hyperinsulinemia stimulates ovarian androgen production, causing ovulatory dysfunction and worsening weight gain, with immune imbalances exacerbating systemic chronic low-grade inflammation (CLGI). Berberine is a natural plant alkaloid that, owing to its multifaceted metabolic effects—including activation of 5′ adenosine monophosphate (AMP)-activated protein kinase (AMPK) and improvement of insulin sensitivity—effectively supports the restoration of homeostasis in women with PCOS. The aim of this narrative review is to comprehensively analyze the metabolic and anti-inflammatory actions of berberine, which, in combination with the known pathomechanisms of PCOS, may constitute a rationale for its therapeutic application. Attention was given to the necessity of actions aimed at increasing the bioavailability of berberine and to the consequences of the fact that berberine, unlike metformin, which is commonly used and has similar properties, is a dietary supplement and not a Food and Drug Administration (FDA)-approved prescription drug.

## 1. Introduction

Metabolic diseases are becoming increasingly common in modern civilizations, and the obesity pandemic is undoubtedly the main reason for their increasing impact on population health, especially on noncommunicable disease burden [1,2,3]. In women of reproductive age, a common health problem associated with reduced fertility or infertility is polycystic ovary syndrome (PCOS), a complex, multisystem disorder characterized by ovarian hyperandrogenism and a broad spectrum of metabolic disturbances that often extend beyond reproductive issues [4,5,6]. While PCOS, one of the most common but treatable causes of infertility in women, can occur in lean patients, typically between 50 and 90% of women with PCOS are either overweight or obese [7,8,9]. Moreover, insulin resistance (IR) is present in 70–80% of patients with PCOS, and >50% of women with PCOS develop type 2 diabetes (T2D) by age 40 [10,11]. A vicious cycle emerges in which IR acts as a core driver of both clinical symptoms and associated obesity, while compensatory hyperinsulinemia stimulates ovarian androgen production, causing ovulatory dysfunction and worsening weight gain [12,13]. These homeostasis disturbances ultimately lead to immune imbalance and dysregulation of inflammatory factors, causing systemic chronic low-grade inflammation (CLGI), which plays a pivotal role in the pathogenesis of PCOS [14,15,16]. Therefore, while traditionally viewed through a reproductive lens, PCOS is now regarded as a comprehensive endocrine–metabolic–immune disorder with prominent features of metabolic inflammation or meta-inflammation [4,5,9,12,13,16]. The growing perception of PCOS as a predominantly metabolic disorder was reflected in the recent recommendation of the European Society of Human Reproduction and Embryology (ESHRE) from 2026, which called for changing the name PCOS to polyendocrine metabolic ovarian syndrome (PMOS) [17].

The interest in natural bioactive compounds with metabolism-modulating properties, particularly antioxidant, anti-inflammatory, antidiabetic, and weight management properties, in the context of their therapeutic use, as well as in the context of PCOS, is constantly growing [18,19]. This also applies to the search for new methods of obtaining extended applications of medicines or increasing the bioavailability of long-known substances, as is the case with berberine [20]. In Asia, the extensive use of this nonbasic and quaternary benzylisoquinoline alkaloid in the form of the stem, stem bark, roots, and root bark of plants rich in berberine, particularly Berberis species, has more than 3000 years of history [21]. These plants, including goldthread (*Coptis sinensis* L.) and barberry (*Berberis* L.), have been foundational in both traditional Chinese medicine (TCM) and Ayurveda, whereas goldenseal (*Hydrastis canadensis* L.), used by Native Americans, has a long history in traditional North American medicine [21,22]. In various regions worldwide, berberine-containing plants are traditionally used to treat, among other diseases, fever as a traditional antipyretic, infections, digestive tract diseases (e.g., diarrhea, bacterial dysentery, and intestinal parasites), respiratory diseases (e.g., influenza), inflammatory disorders, skin diseases, wounds, tumors as a multitarget antitumor agent, eye irritation and surface infections (e.g., conjunctivitis and dry eye), and liver and gallbladder dysfunction [21,22,23,24,25,26,27,28,29,30]. Moreover, berberine has a beneficial effect on metabolism, improving glycemic control by increasing insulin sensitivity in T2D patients and influencing lipid metabolism disorders by significantly reducing total cholesterol and triglyceride levels in blood and hepatic fat accumulation while simultaneously increasing the high-density lipoprotein (HDL or “good”) cholesterol fraction [31,32]. Berberine has also been shown to be involved in the activation of brown adipose tissue (BAT) and stimulation of white-to-brown adipose tissue conversion, which is associated with energy dissipation and weight loss [33]. These metabolic effects provide the basis for testing the usefulness of berberine not only in the treatment of metabolic syndrome and T2D but also in the treatment of PCOS, in which many common metabolic manifestations, such as CLGI, IR, hyperandrogenism, dyslipidemia, and overweight/obesity, are present [34,35].

The aim of this narrative review is to comprehensively analyze the metabolic and anti-inflammatory actions of berberine, which, in combination with the known pathomechanisms of PCOS, may constitute a rationale for its therapeutic applications.

## 2. Berberine

### 2.1. Physicochemical Characterization of Berberine

The phytogenous benzylisoquinoline alkaloid berberine can be isolated from the roots, rhizomes, stems, and bark of many plants, especially plants from the *Berberidaceae*, *Ranunculaceae*, *Menispermaceae*, *Papaveraceae*, and *Rutaceae* families [21,36]. Many traditional low-cost berberine isolation methods (e.g., acidic water extraction, alcohol extraction, alkaline water extraction, and Soxhlet extraction) remain in use, but enhanced extraction rates, reduced extraction times, and minimized solvent consumption have been achieved using advanced, modern, and eco-friendly extraction methods, including ultrasonic-assisted extraction (USE), microwave-assisted extraction (MAE), supercritical fluid extraction (SFE), ultrahigh-pressure extraction (UPE), enzymatic extraction, and deep eutectic solvent (DES) extraction [37,38,39,40].

Berberine is a quaternary ammonium salt with an intensely bitter taste, a cation chemical formula of C_20_H_18_NO_4_^+^, and a molar mass of 336.366 g·mol^−1^ [41,42,43]. The systematic International Union of Pure and Applied Chemistry (IUPAC) name for berberine is 5,6-dihydro-9,10-dimethoxybenzo[g]-1,3-benzodioxolo[6-a]quinolizinium. The alternative name, frequently referred to by the synonym, is 9,10-dimethoxy-5,6-dihydrodioxolo[5-g]isoquinolino[2-a]isoquinolin-7-ium [44].

**Figure 1:**
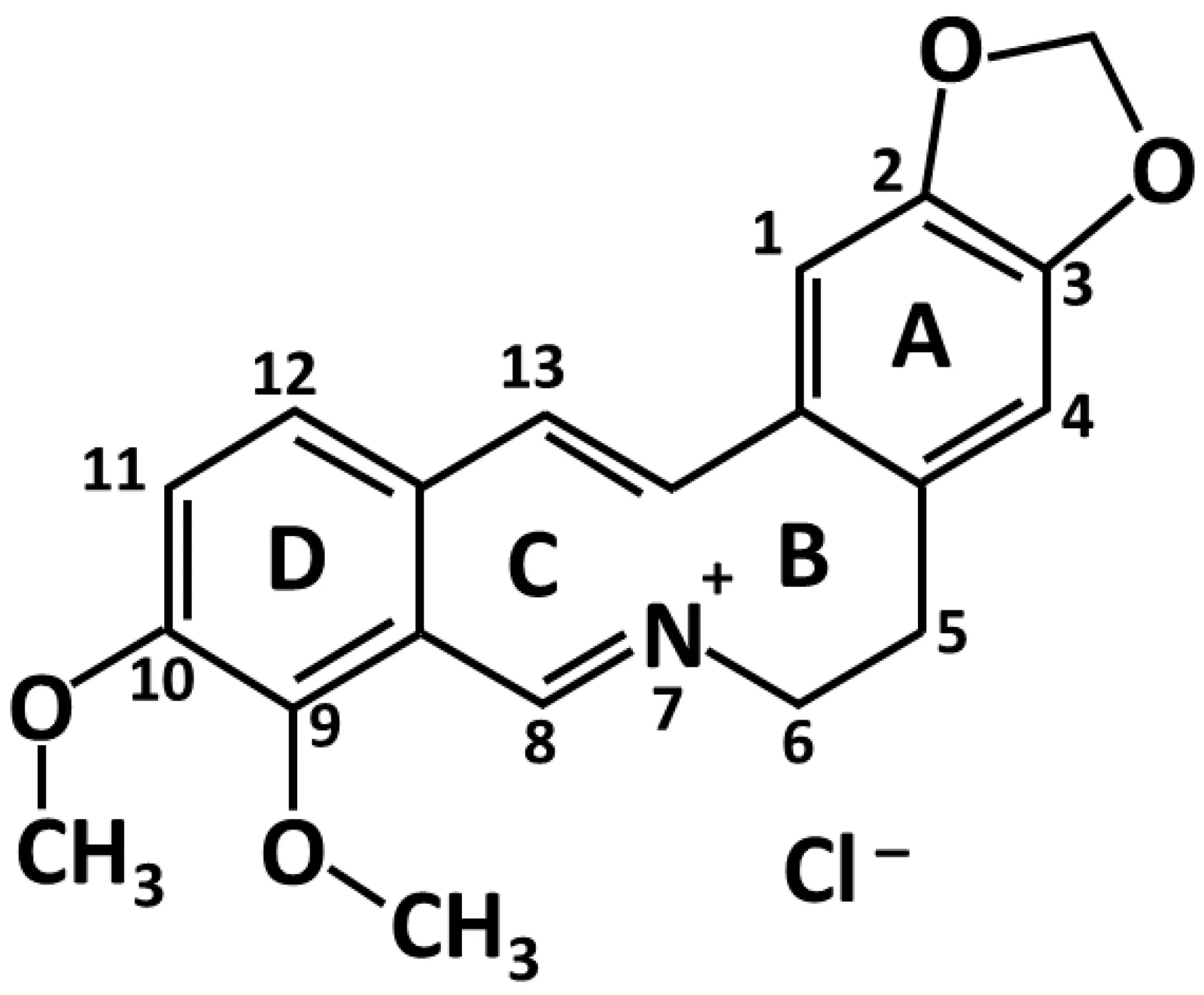
The chemical structure of berberine with four rings (A, B, C, and D) with planar characteristics. International Union of Pure and Applied Chemistry (IUPAC) name: 5,6-dihydro-9,10-dimethoxybenzo[g]-1,3-benzodioxolo[6-a]quinolizinium; chemical formula (the cationic form): C_20_H_18_NO_4_^+^; molar mass: 336.366 g·mol^−1^.

Typically, berberine is marketed as a hydrochloride salt (Figure 1) that forms yellow needle-like crystals because of its high stability and high solubility in water and ethanol; however, some other salts, such as sulfate, phosphate, and bromide, are present [41].

Berberine is insoluble in benzene, ether, chloroform, and other organic solvents [45]. The melting point of berberine, particularly in its common form as berberine chloride, is generally reported to be between 200 and 206 °C (392–403 °F), accompanied by decomposition. While some sources list a melting point range of 145.1–146.7 °C for different forms of berberine, the ~200 °C range is the standard for the hydrochloride hydrate [44]. The fluorescence properties of berberine, with a maximum absorption wavelength of 350 nm and an emission wavelength of 530 nm in 0.01 mol/L sodium dodecyl sulfate solution, can be used to determine its content in TCM products and medicines [46,47].

Carbons C_2_ and C_3_ in ring A of the berberine molecule form a methylenedioxy group responsible for most of the biological activities of berberine, including its anticancer properties, which can be enhanced by C_8_ and C_13_ alkylation (ring C) and/or by modifications at positions N_7_ and C_13_ (ring C), resulting in increased cytotoxicity or enhanced anticellular proliferative activity, respectively (Figure 1) [48,49,50]. The hypoglycemic activity of berberine can be enhanced by alkylation or acylation of the D ring, as well as by introducing cinnamic acid at the 9-O position (D ring) (Figure 1) [20,51,52,53,54]. The presence of a quaternary ammonium structure (with N^+^ in the aromatic ring) in the C ring determines the antibacterial activity of the berberine molecule [47,55].

### 2.2. Biological Activity of Berberine

#### 2.2.1. Spectrum of Tested Therapeutic Uses of Berberine

The wide range of beneficial effects of berberine in disorders of systemic homeostasis involves the function of the immune system, which is manifested by the inhibition of the inflammatory response (anti-inflammatory effects) and its influence on metabolism. This occurs mainly through the activation of 5′ adenosine monophosphate (AMP)-activated protein kinase (AMPK), a key cellular energy sensor responsible for antidiabetic effects through the enhancement of insulin sensitivity and lowering of blood glucose, as well as reducing cholesterol (low-density lipoprotein cholesterol (LDL-C)/triglycerides) and managing fatty liver disease [56,57,58]. The underlying mechanism of these actions involves antioxidant activity, allowing berberine to mitigate oxidative stress-induced damage. Therefore, the therapeutic benefits of berberine treatment are most often considered for cardiovascular diseases, T2D, and metabolic syndrome [47,59,60]. Moreover, by modulating various cell signaling pathways, berberine has anticancer effects through the scavenging of free radicals, modulation of the immune system (e.g., anti-inflammatory effects), induction of apoptosis, cell cycle arrest (inhibition of cell proliferation), inhibition of tumor-induced angiogenesis, inhibition of several key transcription factors, and suppression of metastasis [28,61,62,63,64].

#### 2.2.2. Berberine and the Immune System

The immunomodulatory effect of berberine is bidirectional: on the one hand, it limits the chronic low-grade inflammatory response and hyperactivity of the immune system in autoimmune diseases; on the other hand, berberine chloride enhances innate immunity against pathogens (e.g., viral and bacterial) and reduces the pathogen load, offering protective effects within the intestinal barrier through a healthy microbiome [56,65,66]. Berberine directly suppresses the activity and differentiation of pro-inflammatory CD4+ T-cell subsets, including T helper 1 (Th1) and T helper 17 (Th17) lymphocytes, with subsequent reductions in the production of interferon gamma (IFN-γ), tumor necrosis factor alpha (TNF-α), and interleukin-17A (IL-17A), -17F (IL-17F), -21 (IL-21), and -22 (IL-22) [67,68,69]. Moreover, berberine may indirectly limit Th cell-mediated inflammation by modulating and/or inhibiting other cells involved in CLGI, including autoimmune inflammatory cells, such as regulatory T-cells (Tregs), monocytes and macrophages (e.g., by shifting macrophage polarization from the pro-inflammatory M1 phenotype toward the anti-inflammatory M2 phenotype), and dendritic cells (DCs), including antigen-presenting cells (APCs) [56,70].

The beneficial effect of berberine, an alkaloid of natural origin in CLGI, consists of the inhibition of several key inflammatory signaling pathways, with a subsequent reduction in the biosynthesis of inflammatory cytokines, including interleukin-1 beta (IL-1β), interleukin-6 (IL-6), and TNF-α [59,71,72]. The main pro-inflammatory signaling pathways that are inhibited by berberine are characterized below.

**Nuclear factor kappa-light-chain-enhancer of activated B cells (NF-κB) pathway.** Berberine is recognized as a potent inhibitor of the canonical NF-κB signaling pathway, which is rapid and dependent on NF-κB essential modulator (NEMO)/IκB kinase-γ (IKKγ), and it activates NF-κB (e.g., in the most common form as a dimer of p50/RelA transcription factors) via inhibitory protein kappa B alpha (IκBα) phosphorylation, a critical post-translational modification that leads to IκBα degradation [72]. IκBα degradation is necessary for the activation of the NF-κB transcription factor, allowing its nuclear translocation for the inflammatory response.

Berberine inhibits canonical NF-κB signaling through several mechanisms, including the following:

- **Blocking IκBα degradation.** Berberine prevents the phosphorylation and subsequent degradation of IκBα. By keeping IκBα intact, berberine preserves the NF-κB p50/RelA dimer in an inactive form in the cytoplasm, preventing its translocation into the nucleus [73];
- **Suppressing the phosphorylation of RelA (also known as p65).** Berberine directly reduces the phosphorylation of the NF-κB RelA subunit (Ser536), preventing it from achieving full transcriptional activity [74];
- **Inhibiting IκB kinase (IKK) activation.** Berberine inhibits the activation of IKK, the main enzyme responsible for activation of the NF-κB pathway [75];
- **Upstream signaling interference.** Berberine interferes with upstream pathways that trigger NF-κB, such as the toll-like receptor 4 (TLR4)/myeloid differentiation primary response 88 (MYD88) and the Janus kinase (JAK)/signal transducer and activator of transcription (STAT) pathways [76,77]. The JAK/STAT pathway is inhibited primarily through the suppression of signal transducer and activator of transcription 3 (STAT3) activation [78]. Berberine can also interfere with the IFN-γ/signal transducer and activator of transcription 1 (STAT1)/interferon regulatory factor 8 (IRF8) signaling axis, which is a critical, self-amplifying pathway in the immune system that primarily drives macrophage activation, antigen presentation, and antitumor immunity [79];
- **Sirtuin 1 (SIRT1)-dependent mechanism.** In macrophages, berberine has been shown to suppress NF-κB activation via an SIRT1-dependent mechanism. The upregulation of SIRT1 expression by berberine inhibits the acetylation of the RelA subunit of NF-κB, limiting its nuclear translocation [80]. Through this mechanism, berberine may inhibit macrophage M1 polarization and the production of inflammatory cytokines, particularly in an inflammatory environment [80].

**Suppression of the mitogen-activated protein kinase (MAPK) signaling pathway.** Berberine reduces the production of pro-inflammatory mediators by inhibiting the activation of MAPKs, such as extracellular signal-regulated kinases (ERK1/2), p38 MAPK, and c-Jun NH2-terminal kinases (JNK) [81,82].

**Inhibition of the activation of the nucleotide-binding domain, leucine-rich repeat family, and pyrin domain-containing protein 3 (NLRP3) inflammasome.** Berberine inhibits the activation of the NLRP3 inflammasome, a critical component of the innate immune system, which acts as a sensor for pathogens and cellular damage (danger signals) and is involved in the pathomechanism of CLGI [83]. The downregulation of NLRP3, as well as apoptosis-associated speck-like protein containing a caspase recruitment domain (ASC) and caspase-1, limits the maturation of IL-1β and IL-18 and, consequently, inflammatory cell death (pyroptosis) [84].

**Reducing downstream inflammation within the phosphatidylinositol 3-kinase (PI3K)/protein kinase B (Akt)/mechanistic target of rapamycin (mTOR) pathway.** The PI3K/Akt/mTOR pathway is a critical intracellular signaling network involved in the regulation of the inflammatory response by bridging immune cell activation, metabolism, and cytokine production [85,86]. While often associated with cell growth, its dysregulation is strongly involved in both acute and chronic inflammatory responses and cancer progression [87,88]. Berberine inhibits the activation of PI3K and Akt [89].

**Modulating endoplasmic reticulum (ER) stress-mediated inflammation.** Berberine exerts significant anti-inflammatory effects by alleviating ER stress, a condition in which the accumulation of unfolded/misfolded proteins in the ER triggers cellular dysfunction and inflammation [90,91]. Specifically, in macrophages, berberine inhibits the activation of key ER stress sensors that trigger the unfolded protein response (UPR), such as inositol-requiring enzyme 1 (IRE1), protein kinase R-like ER kinase (PERK), and activating transcription factor 6 (ATF6) [92,93,94,95]. This inhibits the downstream activation of inflammatory pathways related to NF-κB and limits apoptosis in normal healthy cells by inhibiting the expression of the potent transcription factor C/EBP homologous protein (CHOP), also known as DNA damage inducible transcript 3 (DDIT3) or growth arrest and DNA damage-inducible protein 153 (GAGG153) [72,96,97].

**Figure 2:**
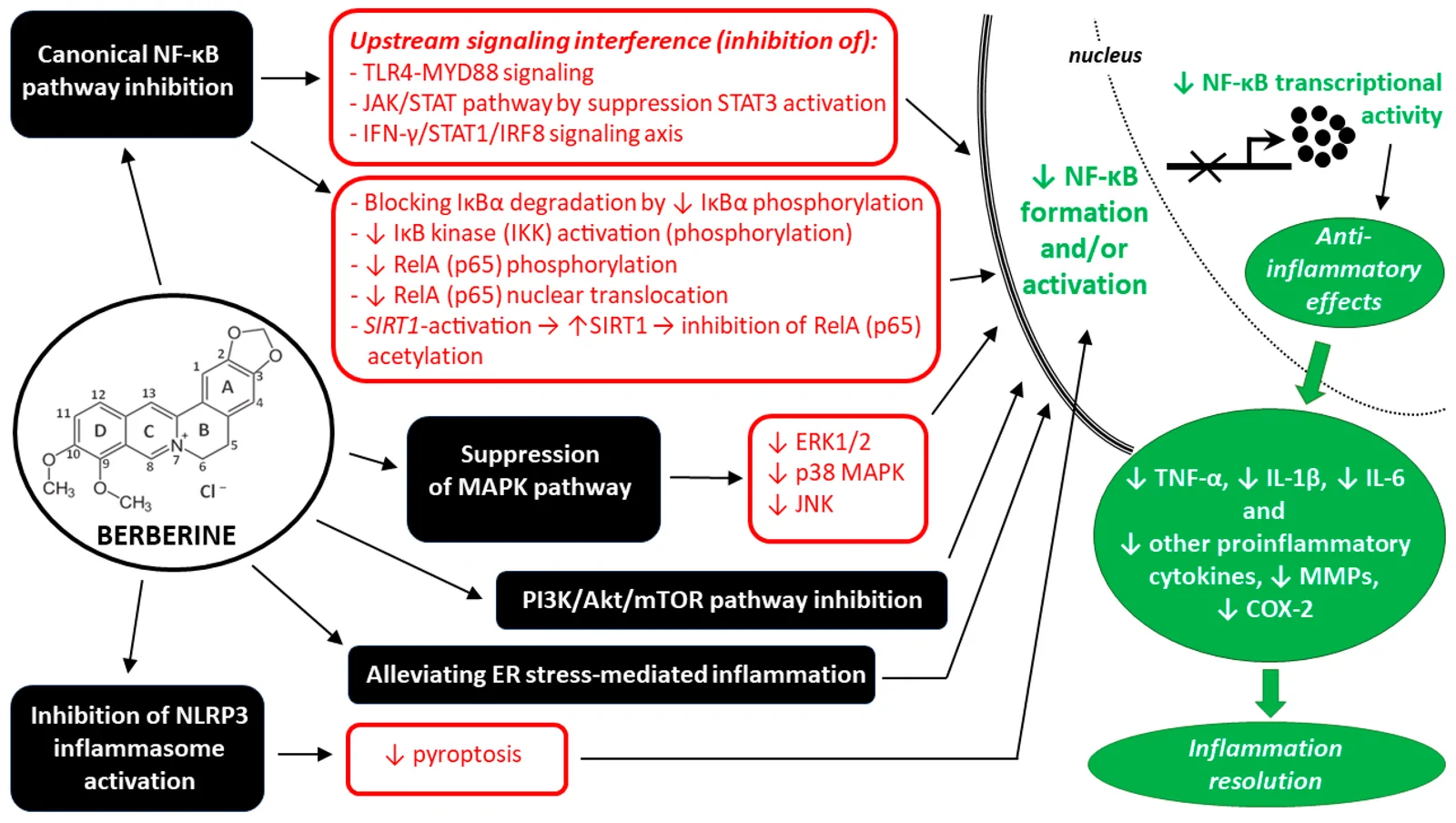
The main NF-kB-dependent signaling pathways affected by berberine during its anti-inflammatory action. Under the influence of berberine, both the synthesis and activation of NF-kB are inhibited. The effects of NF-kB transcription inhibition (marked with a crossed x) are summarized in oval boxes on a green background. Akt—protein kinase B; COX-2—cyclooxygenase 2; ER—endoplasmic reticulum; ERK1/2—extracellular signal-regulated kinases 1 and 2; IκBα (IKKα)—inhibitor of nuclear factor kappa B kinase alpha; IFN-γ—interferon gamma; IL-1β, IL-6—interleukins 1β and 6, respectively; IRF8—interferon regulatory factor 8; JAK—Janus kinase; JNK—c-Jun NH2-terminal kinases, a member of MAPK family; MAPK—mitogen-activated protein kinase; MMPs—matrix metalloproteinases; MyD88—myeloid differentiation primary response 88, an adaptor protein; NFκB—nuclear factor kappa-light-chain-enhancer of activated B cells; NLRP3 inflammasome—nucleotide-binding oligomerization domain (NOD), leucine-rich repeat (LRR)-containing protein (NLR) family member 3 inflammasome; p38 MAPK—a class of mitogen-activated protein kinases (MAPKs); mTOR—mechanistic target of rapamycin; p65(RelA)—transcription factor p65, also known as nuclear factor NF-κB p65 subunit (RelA proto-oncogene); PI3K—phosphatidylinositol 3-kinase; SIRT1—sirtuin 1; *SIRT1*— sirtuin 1 gene; STAT—signal transducers and activators of transcription; STAT1, STAT3—signal transducer and activator of transcription 1 and 3, respectively; TLR4—toll-like receptor 4; TNF-α—tumor necrosis factor alpha. Up and down arrows are typical indications of increase and decrease, respectively.

The signaling pathways presented above, the inhibition of which comprise the essence of the anti-inflammatory effects of berberine, are summarized in Figure 2. Virtually all of them focus on inhibiting NF-κB formation and/or NF-κB activation.

#### 2.2.3. Berberine and the Cardiovascular System

The multifaceted effects of berberine on cardiovascular health are the subject of a growing number of scientific reports [98]. Particular emphasis is placed on the antiatherosclerotic properties of berberine, similar to statins, by reducing the concentration of undesirable lipids (LDL-C and triglycerides) and its cardioprotective effects, including antiarrhythmic effects, lowering blood pressure and improving insulin sensitivity [99,100,101,102].

Atherosclerosis and ischemic heart disease (IHD):

Owing to its antiatherosclerotic effects, berberine limits the development of atherosclerotic plaques in both peripheral and coronary vessels, which reduces the risk of ischemic disease, including coronary heart disease and myocardial infarction [103,104,105]. In the prevention of atherosclerosis, in addition to its influence on lipid metabolism and cholesterol concentration (see the next chapter, Section 2.2.4.), the anti-inflammatory effect of berberine at the level of the intestinal barrier plays an important role [106]. It prevents the secretion of dysfunctional mucus and disruption of the structure of tight junctions (TJs), desmosomes, and adherens junctions (AJs), which consequently helps maintain the integrity of the intestinal barrier. Loss of intestinal barrier function in the form of a so-called leaky gut leads to the penetration of gut bacteria and antigens into the bloodstream, with subsequent activation of systemic inflammation via toll-like receptors (TLRs) and inflammasomes [107,108,109]. Intestinal barrier failure is accompanied by elevated serum lipopolysaccharide (LPS) and inflammatory cytokines, which are correlated with plaque formation and an increased risk of atherosclerotic plaque rupture [110]. In studies on animal models of atherosclerosis, especially in high-fat diet (HFD)-fed mice, berberine restored intestinal barrier tightness, which resulted in increased expression of TJ proteins, increased colonic mucin thickness, and reduced plasma LPS concentrations and levels of pro-inflammatory cytokines, such as IL-1β, IL-6, and TNF-α, in the blood [111,112]. In diabetic rats, berberine reduced inflammation-induced leaky gut conditions via the TLR4/MyD88/NF-κB pathway [113].

In IHD, a significant problem is recurrent states of periodic myocardial ischemia/hypoxia with subsequent restoration of blood flow, known as myocardial ischemia—reperfusion injury (MIRI) [114,115]. This type of damage involves severe metabolic disruptions, including oxidative stress, calcium overload, and inflammation, often causing additional tissue damage by triggering various types of regulated cell death, such as apoptosis, ferroptosis, and autophagy-related cell death [115,116,117]. MIRI may be responsible for approximately 50% of the final infarct size [118]. Studies conducted on isolated cardiomyocytes revealed that pretreatment with berberine reduced MIRI, which was accompanied by a significant reduction in the concentrations of MIRI indicators such as lactate dehydrogenase (LDH) and malondialdehyde (MDA) [119,120]. The cardioprotective effect of berberine against MIRI is derived from a wide spectrum of properties, the most important of which during persistent ischemia are antioxidant, anti-inflammatory, and antiapoptotic capabilities; inhibition of autophagy; promotion of coronary vasodilation; and facilitation of angiogenesis after MIRI [121,122].

Hypertension:

Hypertension is widely recognized as a predisposing factor but also a triggering or accelerating factor for atherosclerosis and IHD [123]. Chronic arterial hypertension, the most significant modifiable cardiovascular risk factor, accounts for 48% of all strokes and 18% of all coronary events [124]. Chronic high blood pressure leads to endothelial dysfunction by damaging the intima, which causes increased permeability to cholesterol (including LDL), the facilitated accumulation of which accelerates the development of atherosclerotic lesions [124,125,126]. The most vulnerable to atherosclerosis are middle- and large-sized arteries, especially the sites of vessel branching [124]. Thickening of the artery walls with loss of elasticity (stiffening) is required to adapt to blood pressure changes [127]. Atherosclerotic plaques that develop during the course of hypertension are less stable and are more susceptible to rupture [128].

Berberine is recognized as a multitarget compound with antihypertensive activity, utilizing several interconnected signaling pathways and mechanisms, including those related to anti-inflammatory and antioxidant properties [129,130,131]. The reduction in blood pressure after the administration of berberine occurs mainly because of the activation of the AMPK–endothelial nitric oxide synthase (eNOS)–nitric oxide (NO) pathway, where AMPK phosphorylates eNOS at Ser1177, leading to increased production of NO with subsequent vasodilation [132,133,134]. Moreover, AMPK signaling causes the vasorelaxation of vascular smooth muscle cells (VSMCs) through a reduction in intracellular calcium [(Ca^2+^)i] because it promotes calcium sequestration via the activation of sarcoplasmic/endoplasmic Ca^2+^-ATPase (SERCA) and activates large-conductance Ca^2+^-activated K^+^ (BK_Ca_) channels [129,135]. Vasoconstriction related to VSMCs is also limited by the inhibitory effect of AMPK on the Ras homolog family member A (RhoA)/Rho-associated protein kinase (ROCK) pathway, which, through its effect on the cytoskeleton, controls cell shape, motility, proliferation, and contraction [136,137].

The suppression of reactive oxygen species (ROS) generation after AMPK activation by berberine within the rostral ventrolateral medulla (RVLM), a key vasomotor center in the brainstem responsible for the control of the sympathetic nervous system, blood pressure, and cardiovascular functions, contributes to a decrease in systemic blood pressure [138,139].

Berberine modulates the activity of the renin–angiotensin–aldosterone (RAA) axis. It acts as a direct angiotensin-converting enzyme (ACE) inhibitor, inhibiting the activity of the enzyme that converts angiotensin I to angiotensin II, a potent pro-inflammatory vasoconstrictor. In animal models, the hypotensive effect of berberine is accompanied by reduced expression of ACE and angiotensin receptor 1 (ATR1) mRNA [133,140,141]. By reducing inflammation, oxidative stress, and ER stress, berberine limits podocyte damage induced by excess aldosterone [142].

Berberine modulates the activity of the gut microbiota within the gut–kidney axis and reduces inflammation, which occurs mainly through a mechanism known as the gut flora–short-chain fatty acid (SCFA)–LPS–IL-6–STAT3 axis. This leads to a decrease in blood pressure in the circulatory system, with an accompanying increase in SCFA production by beneficial bacteria and a reduction in LPS production and signaling via STAT3 due to a reduction in pro-inflammatory bacteria in the gut [143,144].

#### 2.2.4. Berberine and Glucose and Lipid Metabolism

The metabolic effects of berberine facilitate the maintenance of a healthy body weight, as suggested by the results of some studies reporting a reduction in body mass index (BMI) [145].

Glucose metabolism:

In terms of its effect on glucose metabolism, berberine is similar to metformin, as it activates AMPK, increases glucose uptake, and improves insulin sensitivity, which translates to a reduction in homeostatic model assessment of insulin resistance (HOMA-IR) values [146,147,148]. Berberine protects pancreatic β-cells from dysfunction and apoptosis by reducing oxidative stress and inflammation, ultimately preserving insulin secretory function [149,150]. It increases glucose consumption and uptake in muscles and adipocytes [151]. For example, berberine stimulates glycolysis in peripheral tissues by inhibiting mitochondrial respiratory chain complex I, forcing cells to rely on glycolytic pathways [151]. The observed effects of lowering fasting blood glucose, 2 h post-prandial blood glucose, or normalization of the 75 g oral glucose tolerance test (OGTT) result and reducing IR in individuals with metabolic disorders are often comparable to those of conventional antidiabetic medications (e.g., metformin) [146,152,153].

Lipid metabolism:

In addition to its effects on blood sugar, by being an effective, safe, and natural lipid-lowering agent, berberine helps improve triglyceride and total cholesterol levels [154]. The effect of berberine on lipid metabolism manifests itself as a comprehensive modulation of multitarget pathways in the liver, intestine, and vascular system, the effects of which in hyperlipidemia, obesity, and nonalcoholic fatty liver disease (NAFLD) are, according to some studies, comparable to those of conventional treatments such as metformin or statins [31]. Berberine promotes intestinal growth of beneficial bacteria such as *Akkermansia* while reducing bacteria that contribute to fat accumulation and inflammation [155]. By inhibiting acetyl-CoA-cholesterol acyltransferase 2 (ACAT2), berberine limits intestinal cholesterol absorption [156,157]. Additionally, the reduction in blood lipid levels is supported by the increased synthesis of SCFAs (e.g., butyrate) under the influence of berberine [144,158]. Fat uptake in the liver is also reduced by the modulation of bile acid metabolism by berberine, which is manifested by the inhibition of bile salt hydrolase (BSH), with a subsequent increase in conjugated bile acids and the activation of the nuclear farnesoid X receptor (FXR) [159]. Activation of FXR primarily acts as a sensor for bile acids, leading to the inhibition of bile acid synthesis, reduced hepatic lipid production, increased bile acid transport, and improved glucose metabolism [160]. The beneficial changes observed in the lipid profile after berberine supplementation included a reduction in the levels of total cholesterol (TC), LDL-C, apolipoprotein B, and triglycerides and an increase in the lipid fraction of high-density lipoprotein cholesterol (HDL-C) [99,161,162].

#### 2.2.5. Anticancer Effects of Berberine

Research on berberine as a cancer treatment has grown over the last 20 years [163,164]. Berberine’s anticancer effects on breast, lung, stomach, and liver tumors are proven in lab and animal studies [29,62].

Key mechanisms of berberine’s anticancer activity include:

- **inducing apoptosis** by increasing the expression of pro-apoptotic proteins (e.g., Bax) and reducing the expression of antiapoptotic proteins (e.g., Bcl-2) [165,166,167];
- **promoting cancercell cycle arrest** in the G0/G1 or G2/M phase via regulatory protein modulation like p53, p21, and cyclins [166];
- **blocking key survival pathways** like PI3K/Akt/mTOR, MAPK/ERK, and Wnt/β-catenin that stops cancer cells from growing and surviving [28,168,169];
- **inhibiting cancer spread (metastasis) and tumor angiogenesis** by downregulating invasive enzymes, such as matrix metalloproteinases (MMP-2 and MMP-9), and suppression of the epithelial–mesenchymal transition (EMT) with simultaneous downregulation of vascular endothelial growth factor (VEGF) [170,171,172,173].

In addition, berberine may fight cancer by cleaning cells (activating autophagy), balancing gut bacteria, and boosting other drugs [163].

However, the key to achieving effective berberine therapy in clinical settings is to consider the histological and molecular diversity within the same types of cancer (intratumoral heterogeneity) in individual patients [164,174,175,176]. The most significant clinical confirmation to date is its efficacy in reducing the recurrence of colorectal adenomas [177]. An extended follow-up of a previous clinical trial (NCT02226185) during the post-treatment observational phase revealed that the protective effects of berberine persisted for at least 6 years after treatment cessation, with a lower adenoma recurrence rate in the berberine-treated group than in the placebo control group (34.7% vs. 52.1%) and a lower neoplasm occurrence rate (63.4% vs. 71.0%) [177].

### 2.3. Bioavailability of Berberine

The therapeutic use of the beneficial effects of berberine is severely limited by its very low absolute oral bioavailability, which is usually estimated at less than 1% [178]. Low intestinal absorption is enhanced by the poor water solubility of berberine [179,180]. Low plasma levels of berberine are also caused by extensive intestinal first-pass elimination and the predominant hepatic distribution of this compound [181]. Intestinal first-pass elimination has been shown to be a much more significant barrier than hepatic metabolism because, according to various estimates, as much as approximately 50–75% of absorbed berberine is metabolized by enterocytes before reaching the bloodstream [47,181]. Additionally, absorption in small intestinal epithelial cells by passive diffusion is limited by the presence of ATP-dependent transporter proteins in the form of P-glycoprotein (P-gp) efflux pumps, which are responsible for the active transport of berberine back into the intestinal lumen [182,183]. In phase II of berberine metabolism in enterocytes, its major metabolites are formed through glucuronidation and sulfation [184,185]. The high metabolism of berberine suggests that while its plasma levels remain low, its pharmacological effects are achieved through high local concentrations within intestinal tissues or through its active metabolites [181].

A significant improvement in the bioavailability of berberine is achieved when it is administered intravenously, but in clinical practice, this is discouraged by the relatively high risk of serious side effects, including respiratory arrest [186]. Therefore, attempts are being made to increase the bioavailability of oral berberine by using P-gp inhibitors (e.g., silymarin) or increasing the permeability of the intestinal barrier, as well as by formulating nanoparticles (e.g., nanosuspensions of polymer–lipid hybrid nanoparticles loaded with berberine phospholipid complexes), micelles (e.g., selenium-coated nanostructured lipid carriers), or solid dispersions to carry berberine doses [183,186,187,188]. In vivo, the bioavailability of relatively small berberine hydrochloride molecules increases after their conversion into organic salt compounds, especially berberine fumarate and berberine succinate [189]. To improve bioavailability, attempts have also been made to increase hydrophobicity by alkylation at positions C5–C9 with simultaneous enhancement of lipophilicity (e.g., after 9-O benzylation of the berberine molecule) [49,50,186,190]. For instance, compared with standard berberine hydrochloride, berberine phytosomes—which bind the compound to phospholipids—have been shown to increase absorption by up to 10 times [191].

The most commonly studied dose of berberine hydrochloride in PCOS trials is 500 mg three times daily, taken with meals, for a total daily dosage of 1500 mg [192]. Such a dose appears to strike a balance between efficacy and tolerability. Berberine should be taken with meals to improve its absorption and reduce the risk of gastrointestinal side effects (mild gastrointestinal upsets, including constipation, temporary diarrhea, cramping, and bloating), which are the most common complaints [192].

## 3. PCOS

**Figure 3:**
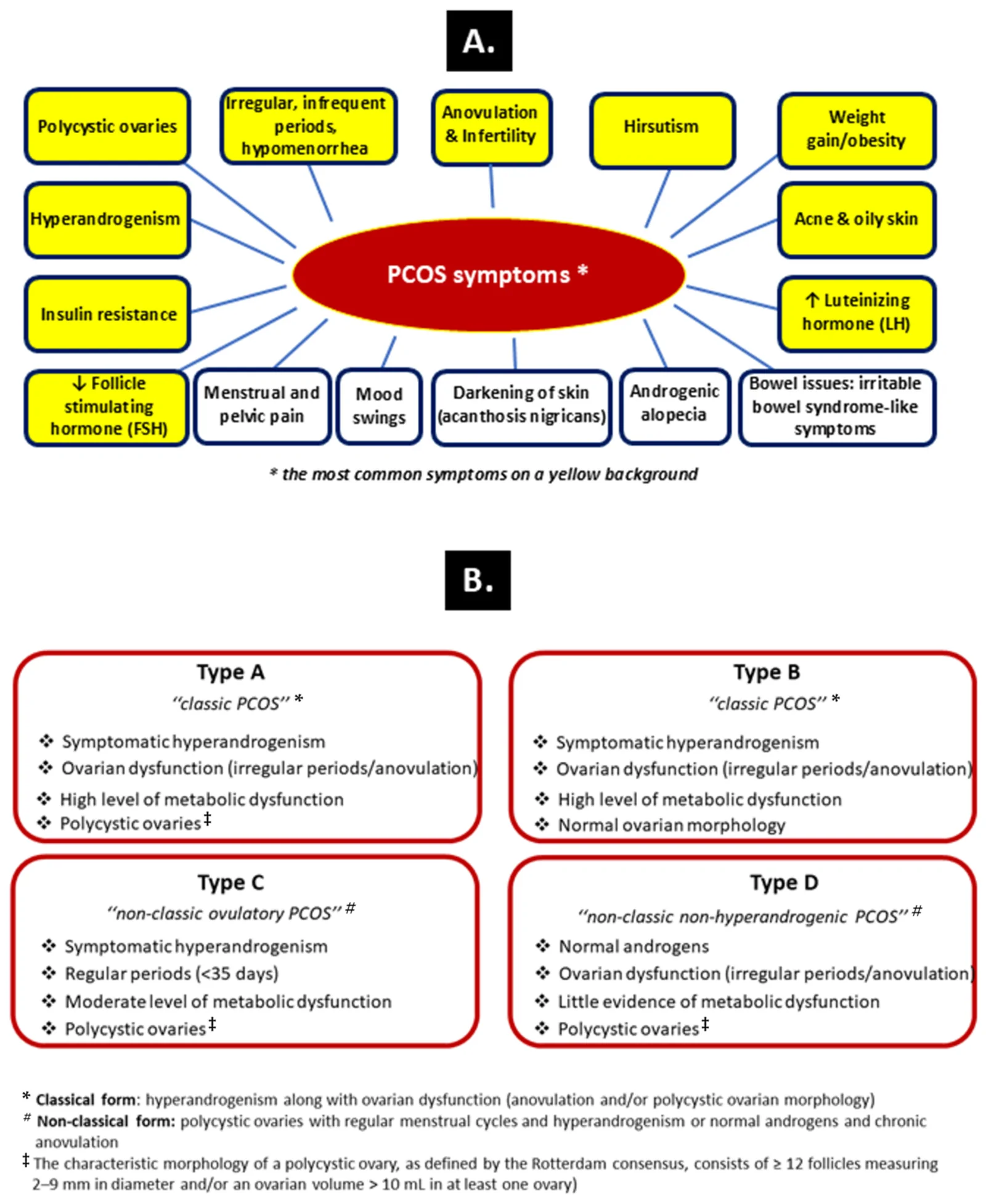
Symptoms of PCOS (**A**) and the simple clinical classification taking into account the phenotypes of PCOS (**B**) [205]. Adopted from [15]. Up and down arrows are typical indications of increase or decrease, respectively.

Polycystic ovary syndrome (PCOS), originally known as Stein–Leventhal syndrome, was first described by Irving Freiler Stein and Michael Leo Leventhal in 1935 [193]. PCOS is among the most common hormonal disorders among women of reproductive age, affecting 4–20% of this population globally [194,195]. The unusually wide range of reported PCOS prevalence reflects the highly heterogeneous nature of this complex disorder, the occurrence of which is the result of the interaction of genetic, environmental, endocrine, and behavioral factors [196,197,198]. Common and major symptoms of PCOS are derived from the effects of systemic androgen excess and include anovulation leading to fertility issues, typical polycystic ovarian morphology with small, fluid-filled follicles (cysts), absent or scanty, irregular menstrual bleeding, inflammatory skin lesions in which the skin’s sebaceous glands become clogged and infected, known as acne, and hirsutism, which is excessive growth of dark or coarse hair in a male-like pattern—face, chest, and back [199,200]. The secondary manifestations include multiple metabolic, cardiovascular, and psychological disorders (Figure 3A) [201,202,203,204].

Owing to significant gaps in the understanding of the pathomechanisms of PCOS, this syndrome remains underdiagnosed, and the treatment of female patients is delayed, which affects outcomes [206]. Of particular importance here is the lack of timely implementation of therapeutic strategies for long-term health issues known as PCOS-related complications, such as IR, among metabolic abnormalities [207].

The phenotypes of PCOS can be subdivided into four different types according to simple clinical classification (Figure 3B). There are two “classic phenotypes” of PCOS, in which a high level of metabolic dysfunction is observed (A—with polycystic ovaries; B—with normal ovarian morphology), and two “nonclassic” types of PCOS: C—with preserved ovulation; D—without hyperandrogenism [205]. The course of PCOS characteristically evolves with age, from a reproductive disease to a more metabolic disorder with an increased incidence of type 2 diabetes and cardiovascular disease (e.g., atherosclerosis and high blood pressure) later in life [206,208]. The multifactorial etiology of PCOS, with the presence of different genetic variants that play crucial roles in its pathogenesis, is also accompanied by strong epigenetic and environmental influences, including dietary and lifestyle factors [15,209].

### Metabolic Disorders in PCOS Patients

As mentioned above, phenotype A, often also called the PHO phenotype (P—polycystic ovaries, H—hyperandrogenism, O—ovarian dysfunction with oligo/anovulation), which is the classic form of PCOS, is associated with the highest metabolic risk, which in clinical practice indicates the greatest severity and diversity of metabolic disorders [210,211,212,213,214]. With respect to fertility, in vitro fertilization (IVF) studies have shown that although women with the PHO phenotype may produce a high number of eggs, their quality may be lower, which may result in a higher risk of pregnancy loss in the second trimester than in women with milder PCOS phenotypes [215].

The cluster of metabolic dysfunctions observed in PCOS largely fits the picture of metabolic syndrome, with hyperandrogenism interacting with insulin pathways being responsible for a self-perpetuating vicious cycle of metabolic and reproductive dysfunction [211]. In particular, the interaction between high androgen levels and hyperinsulinemia caused by IR leads to the following consequences:

- Hyperandrogenism directly contributes to IR by promoting abdominal adiposity (visceral fat), increasing free fatty acids (FFAs) and inhibiting glucose transporter type 4 (GLUT4) in adipose tissue, among other effects, through overexpression of miRNA-93 [212,213,214];
- Hyperinsulinemia acts directly on ovarian thecal cells and adrenal cells in the *zona reticularis* to stimulate androgen (testosterone and androstenedione) production [216,217]. Moreover, excess insulin also suppresses the production of sex hormone-binding globulin (SHBG) in the liver, increasing the amount of free, bioavailable, and active androgen [218,219];
- Ovarian dysfunction with anovulation and infertility occurs because of the formation of multiple small atretic ovarian follicles (polycystic morphology) rather than the development of an ovulating mature oocyte [220,221]. The combined antiovulatory effects of elevated insulin and androgen levels also cause scant, irregular, or absent menstrual bleeding (oligomenorrhea/amenorrhea) [222,223];
- Systemic metabolic disorders with CLGI, a persistent, silent state of immune system activation characterized by elevated inflammatory markers [e.g., high-sensitivity C-reactive protein (hs-CRP), IL-1β, IL-6, TNF-α, VEGF, ferritin, erythrocyte sedimentation rate (ESR), white blood cell count (WBC) and plasma viscosity (PV)] coexist with oxidative stress, dyslipidemia (high triglycerides and LDL-C, low HDL-C), NAFLD, and increased risk of T2D [34,220,224,225]. CLGI is often accompanied by vitamin D deficiency and altered gut microbiome composition (increased levels of pro-inflammatory bacteria) [226,227]. The pro-inflammatory ovarian and systemic environment is accompanied by a significant increase in the ratio of M1 to M2 macrophages [228].

**Figure 4:**
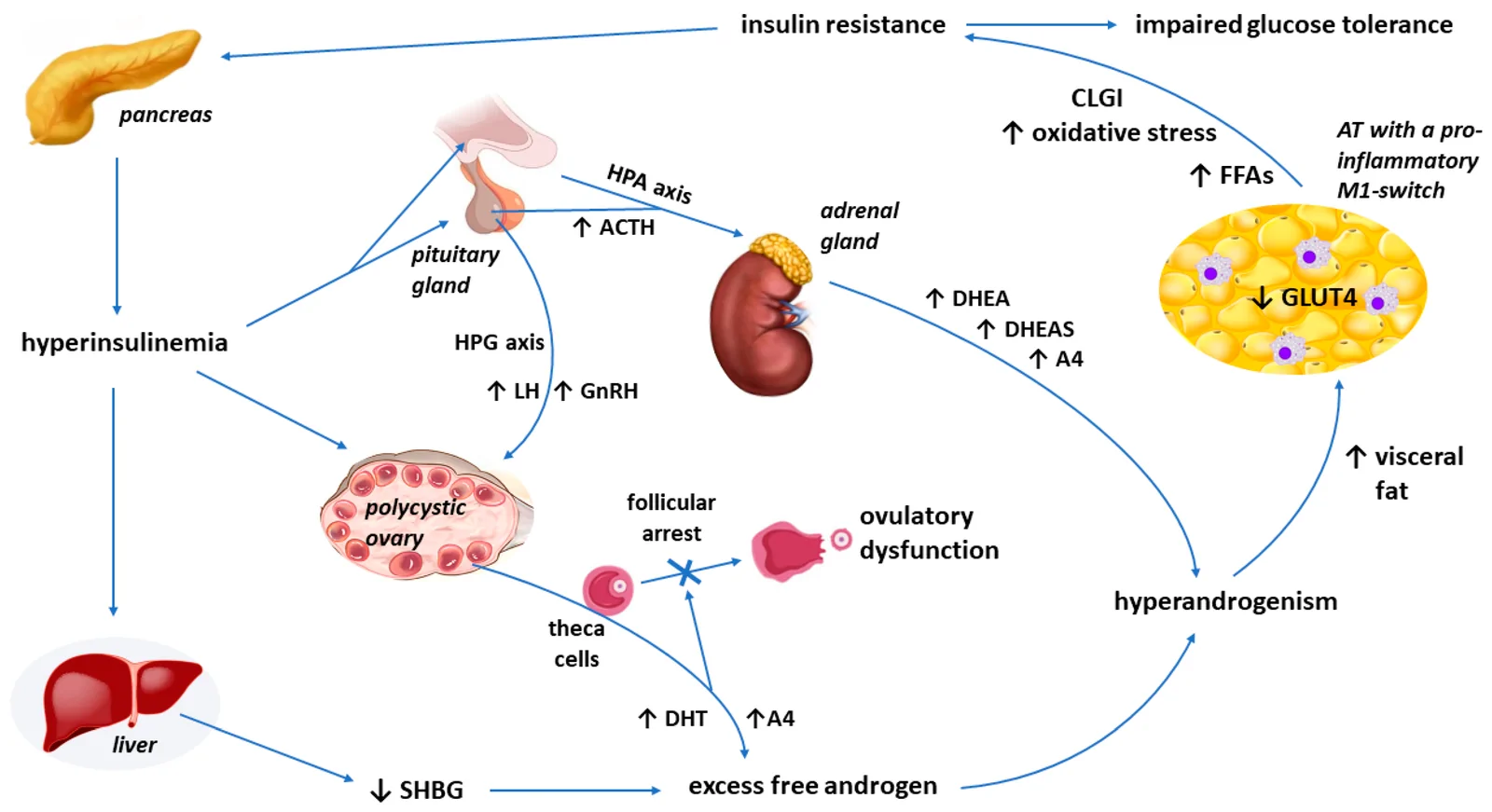
The vicious cycle mechanism in the pathogenesis of PCOS [15,34,211,213,220,229]. Impaired glucose tolerance due to insulin resistance [IR] leads to hyperglycemia and stimulates the β-cells of the pancreatic islets to overproduce insulin. Hyperinsulinemia has a multifaceted effect, leading to a reduction in the production of sex hormone-binding globulin (SHBG) in the liver, and by acting on the hypothalamic–pituitary–gonadal (HPG) axis and often also on the hypothalamic–pituitary–adrenal (HPA) axis, it leads to hyperandrogenism. Hyperandrogenism causes follicular arrest and ovulatory dysfunction, and by promoting abdominal adiposity, disturbing glucose transport in adipose tissue (AT), and increasing the release of free fatty acids (FFAs) by stimulating lipolysis, it increases IR. Hyperandrogenism stimulates inflammation in AT with a shift towards pro-inflammatory classically activated macrophages (M1). This is accompanied by an increase in the level of oxidative stress and systemic chronic low-grade inflammation (CLGI). A4—androstenedione; ACTH—adrenocorticotropic hormone; DHEA—dehydroepiandrosterone; DHEAS—dehydroepiandrosterone sulfate; DHT—dihydrotestosterone; GnRH—gonadotropin-releasing hormone; LH—luteinizing hormone. Up and down arrows are typical indications of increase or decrease, respectively.

Essentially, hyperandrogenism and IR act as both a cause and a consequence of each other, where IR stimulates androgen production and androgens aggravate IR [229]. The importance and mutual interactions of hyperandrogenism and IR in the pathophysiology of PCOS and accompanying metabolic disorders are presented in Figure 4.

Another vicious cycle mechanism is associated with hyperandrogenism, which leads to a disturbance in the proportion of secreted gonadotropins, follicle-stimulating hormone (FSH), and luteinizing hormone (LH) in PCOS [230]. Approximately 65.5% of PCOS patients have elevated basal LH levels [231]. This leads to hyperstimulation of theca cells in the ovary, promoting excessive androgen production [232,233]. Elevated androgens directly impair the sensitivity of the hypothalamus to progesterone-mediated negative feedback, which is necessary to slow the GnRH pulse generator [234]. Additionally, reduced progesterone feedback in PCOS is caused by anovulation, i.e., a lack of the corpus luteum, the main source of progesterone in the second half of the cycle [235]. Under these conditions, dysfunction of KNDy (kisspeptin, neurokinin B, dynorphin) neurons in the arcuate nucleus (ARC), constituting the “GnRH pulse generator”, occurs [236]. Increased activity of KNDy neurons in PCOS particularly disrupts the proper formation of excitatory GABAergic signaling to GnRH neurons, with rapid GnRH pulses preferentially promoting the production of LH over FSH, creating a self-perpetuating cycle of androgen excess and follicular arrest [237]. In a prenatally androgenized (PNA) sheep model, an animal model of PCOS, a significant increase in the density of GABAergic contacts onto GnRH neurons arises from progesterone receptor-containing cells in the ARC, which are part of the KNDy population [238].

## 4. Therapeutic Potential of Berberine in PCOS Patients

Comparison of the beneficial biological effects of berberine in the human body, which allows for the restoration/maintenance of homeostasis in a wide range of conditions through its influence on the immune system (anti-inflammatory effect and strengthening innate immunity against pathogens), intestinal microbiome, glucose metabolism (antidiabetic and insulin-sensitizing effects), lipid metabolism (reduction in cholesterol, improvement in lipid profile, management of fatty liver disease), and circulatory system (antiatherosclerotic, cardioprotective effects including antiarrhythmic and hypotensive effects), or even its anticancer effects, with pathomechanisms in PCOS, provides a rationale for the implementation of berberine in the treatment of PCOS [239,240]. The metabolic effects of berberine, which are primarily derived from limiting cell damage due to oxidative stress, fit into the spectrum of disorders accompanying PCOS [239]. To demonstrate the therapeutic potential of berberine in PCOS, the mechanisms underlying the beneficial effects of berberine in PCOS-related metabolic/hormonal disorders are summarized in Table 1**.**

**Figure 5:**
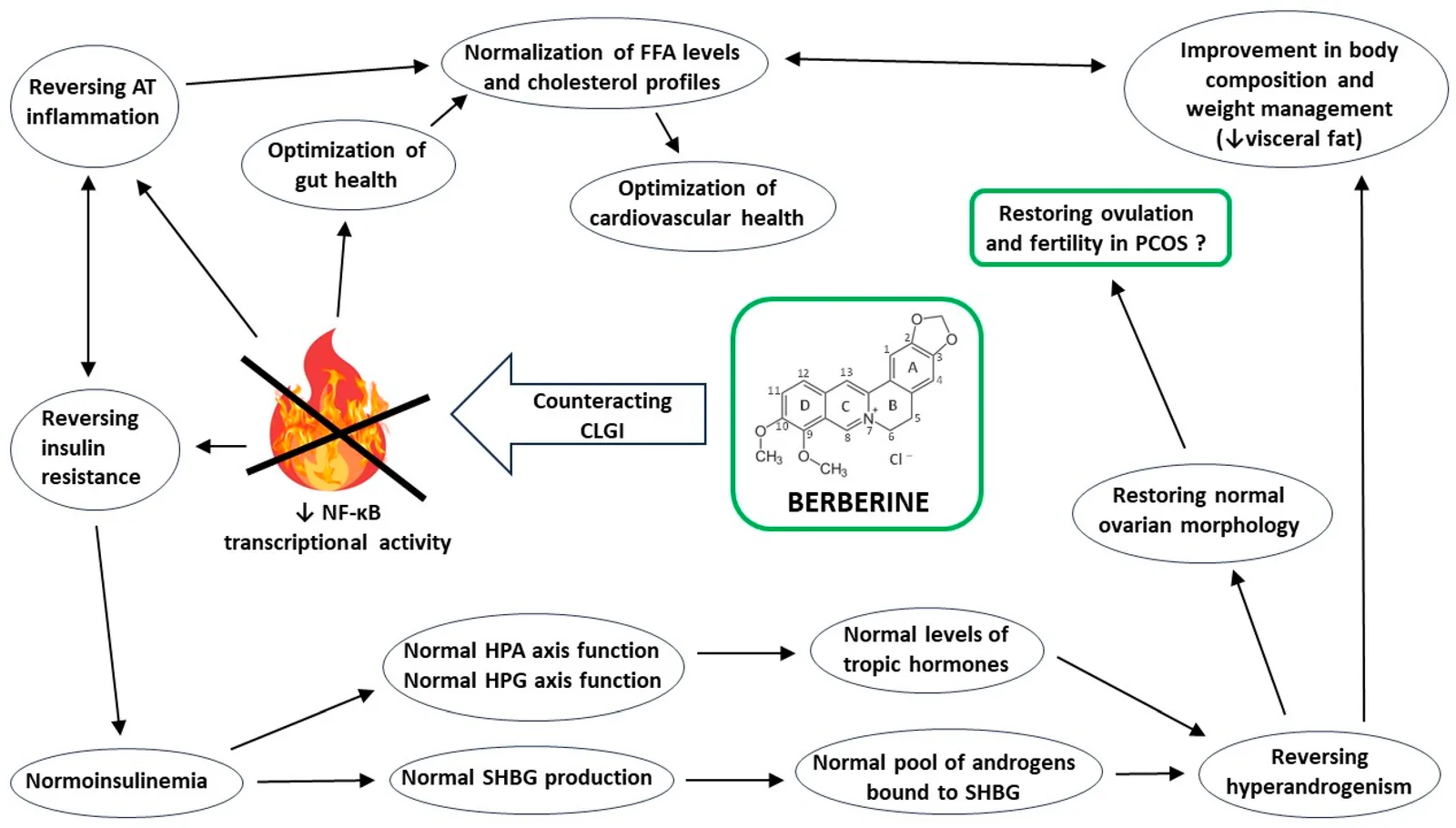
Potentially beneficial therapeutic effects of berberine taking into account the pathomechanisms of PCOS. Counteracting the development of chronic low-grade inflammation (CLGI) by reducing the transcriptional activity of nuclear factor kappa-light-chain-enhancer of activated B cells (NF-κB) plays a key role in reversing insulin resistance and inflammation in adipose tissue (AT) and at the level of the intestinal microbiome. Achieving normoinsulinemia contributes to the restoration of normal function of the hypothalamic–pituitary–adrenal (HPA) axis, hypothalamic–pituitary–gonadal (HPG) axis, and to an increase in the production of sex hormone-binding globulin (SHBG) to the normal level. Symptoms of hyperandrogenism disappear, with a reduction in visceral fat accumulation, improvement in body composition and weight control, return to normal ovarian morphology (cyst disappearance), and an increased likelihood of ovulatory cycles, creating a chance for fertilization (?—effect uncertain). Additionally, normalization of free fatty acid (FFA) levels and cholesterol profiles has a beneficial effect on cardiovascular health. Double-headed arrows indicate mutual interactions. Down arrows are typical indications of increase or decrease.

The above potentially beneficial effects of berberine therapy with regard to the pathomechanisms of PCOS are summarized in Figure 5.

Owing to the highly heterogeneous nature of PCOS, the therapeutic efficacy of berberine varies significantly. Berberine has shown significant therapeutic potential for treating PCOS, primarily by affecting IR, which is a core driver of this condition. Therefore, the greatest efficacy of berberine should be expected in classic forms of PCOS (type A and type B; see Figure 3B) characterized by a high level of metabolic dysfunction [255].

When analyzing the target population for the use of berberine in PCOS, i.e., women of reproductive age who may become pregnant or are often treated for infertility, it should be remembered that the use of berberine is generally not recommended during pregnancy due to the possibility of inducing uterine contractions increasing the risk of miscarriage, as well as the lack of complete data on the safety profile [270]. Berberine should also not be used during breastfeeding because it passes into breast milk and increases bilirubin levels in a newborn’s blood. Hyperbilirubinemia can cause a dangerous condition called kernicterus, which leads to brain swelling and severe brain damage in newborns [270,271].

*Clinical evidence of berberine’s effectiveness in PCOS.*

Despite numerous clinical trials aimed at utilizing the biological properties of berberine, primarily its effect on metabolism due to hypoglycemic and anti-inflammatory properties, systematic reviews and meta-analyses indicate that clinical evidence regarding its effectiveness for core reproductive outcomes in PCOS remains weak, low-quality, and limited [247,249,260]. There is a lack of reliable data confirming the effectiveness of berberine in improving the endpoints (target results) of clinical trials, such as conception rate, live birth rate, or pregnancy outcomes [272]. Analyzing the current data, it can be assumed that berberine provides no statistically significant increase in live birth or ovulation rates compared to placebos or standard metformin therapy [192,273]. Most randomized controlled trials assessing berberine feature very small cohorts [247,273]. Therefore, small sample size limits the statistical power needed to generalize results to wider, diverse populations. Additionally, the interpretation of results is complicated by the high degree of heterogeneity of the samples [273]. Available clinical trials are highly diverse in terms of demographic characteristics, standard baseline treatments, and overall study designs [249]. To date, most of the studies have been performed in Chinese subjects, raising the possibility of a high risk of selection bias and the need to confirm the validity of these results also in subjects of other ethnic origins [251,274]. The key flaws of the research on berberine in PCOS also include the short duration of the study. Most tracked medical trials only span 3 to 6 months [247]. There is an absence of long-term data regarding the sustainability of symptom management or safety profile changes over multiple years. This situation may, on the one hand, lead to an underestimation of the possible impact of berberine on some metabolic pathways that may require a longer intervention to show beneficial effects, as well as to establish a possible beneficial effect on clinical outcomes in PCOS. On the other hand, the safety and tolerability of berberine may be overestimated [275]. Altogether, the above facts support the implementation of long-term studies in large populations, which can guarantee the final determination of the possible clinical beneficial effects of berberine in PCOS as well as its long-term safety [247,249,260].

*Berberine versus metformin*.

Owing to its ability to activate the AMPK signaling pathway—often referred to as the body’s “metabolic switch”—berberine is frequently mentioned alongside metformin and compared to it as “nature’s metformin”. However, the properties, mechanisms of action, potencies, and safety profiles of a plant-derived dietary supplement (berberine) differ significantly from those of a prescription pharmaceutical (metformin) [276]. The difference in bioavailability is particularly striking: the intestinal absorption of metformin reaches 50–60%, whereas that of berberine does not reach 1% [178,276]. The poor absorption of berberine in the gastrointestinal tract, its extensive breakdown by intestinal bacteria, and its rapid metabolism in the liver necessitate the use of much higher oral doses to achieve clinical effects comparable to those of a lower dose of metformin [192].

Despite this massive difference in absorption, clinical trials have shown that the blood sugar-lowering efficacy of berberine, such as a reduction in glycosylated hemoglobin (HbA1c), is surprisingly comparable to that of metformin [277]. This is because metformin primarily limits glucose production in the liver, whereas berberine acts on multiple pathways, including increasing insulin secretion and modulating the gut microbiota to inhibit intestinal glucose absorption. The multidirectional action of berberine, starting at the intestinal level, largely compensates for its poor absorption into the systemic circulation [276,278].

The two substances differ in the speed at which distinct therapeutic effects appear. Metformin typically acts quickly—within 4 to 5 days—whereas effects comparable to those of berberine are observed only after several months of consistent use [279].

Berberine has a more profound, favorable effect on cholesterol (lowering LDL-C and triglycerides) than metformin does because, unlike metformin, it is an inhibitor of proprotein convertase subtilisin/kexin type 9 (PCSK9), a secreted protein and a critical regulator of LDL-C by inducing degradation of the LDL receptor (LDLR) within the hepatocyte lysosome. Reduced expression of PCSK9 significantly improves the survival rate of cardiovascular disease (CVD) patients [280]. Metformin primarily targets blood glucose with only minimal, indirect lipid-lowering effects.

Berberine and metformin exhibit similar side effects, which manifest primarily as gastrointestinal disorders (diarrhea, stomach cramps or abdominal pain, bloating, and increased gas and nausea) [281]. Berberine is often reported to have a milder gastrointestinal side effect profile [282]. Chronic use of both berberine and metformin can occasionally lead to vitamin B12 and iron deficiencies [283]. A metallic taste in the mouth or a loss of appetite may sometimes occur after taking metformin. In addition to being associated with metformin-related gastrointestinal issues, berberine can sometimes cause constipation [192].

Berberine strongly inhibits several liver enzymes, including the major cytochrome P450 (CYP) enzymes (i.e., CYP3A4, CYP2D6, and CYP2C9) that metabolize approximately 70–80% of all prescription medications, possibly altering the effectiveness of some medications or increasing their toxic effects [284]. Considering that CYP3A4 substrates include statins, blood pressure drugs, and antianxiety pills, CYP2D6 substrates include antidepressants and pain relievers, and CYP2C9 substrates include anticoagulants like warfarin and anti-inflammatory drugs (e.g., ibuprofen), the effects of co-therapy with berberine may be serious [284]. Additionally, berberine causes inhibition of P-glycoprotein, which acts as an ATP-dependent transmembrane efflux pump that actively transports toxins, drugs, and foreign substances out of cells. Consequently, inhibition of P-glycoprotein increases drug bioavailability, which leads to higher absorption, greater tissue entry, and a higher risk of side effects including neurotoxicity attributable to compromised blood–brain barrier function [285]. Therefore, taking berberine concurrently with prescribed antidiabetic medications poses a risk of hypoglycemia due to the synergistic glucose-lowering effects [277]. Metformin, which is eliminated via renal clearance, has a safer drug interaction profile; however, its use requires ensuring the absence of specific contraindications—primarily impaired kidney function—to avoid the dangerous complication of lactic acidosis [286].

With respect to long-term use safety, it is worth remembering that berberine is a supplement and that its purity can vary by brand. Metformin, on the other hand, is strictly regulated globally and is a Food and Drug Administration (FDA)-approved prescription medication with more than 60 years of documented cardiovascular and safety data [287].

While some short-term studies have shown that the efficacy of berberine is similar to that of metformin in lowering blood sugar levels, experts emphasize that these compounds are not directly interchangeable. Moreover, the hypoglycemic effects of berberine have not been extensively analyzed in the long-term treatment of diabetes [288]. From a clinical perspective, compared with metformin, berberine may have greater potential to reduce the risk of cardiovascular disease in patients with PCOS because of its effects on lipid metabolism and hormonal balance, including its antiandrogenic effects and modulation of the gut microbiota [278,289]. Berberine shows promise for multiple metabolic markers, which makes its potential benefits go beyond blood sugar regulation, especially when combined with lifestyle interventions [281]. Therefore, berberine is superior to metformin in alleviating hyperlipidemia and obesity [192,290].

## 5. Concluding Remarks

Through trial and error, ancient civilizations identified biologically active substances of natural origin that remain the foundation of medicine to this day. A detailed understanding of the mechanisms of action of substances such as berberine—known from ancient herbal traditions—began with the development of modern medicine, including chemistry-driven pharmacology, as well as physiology and pathophysiology. The multifaceted actions of berberine—including anti-inflammatory and metabolic effects achieved through the regulation of carbohydrate, lipid, and hormonal balance—are attracting particular interest from researchers and clinicians in the development of therapeutic approaches for PCOS. The metabolic, hormonal, and immunological disorders that coexist in PCOS create a vicious cycle of disease, consisting of a self-sustaining loop among IR, hyperinsulinemia, and hyperandrogenism. Additional amplification cycles in PCOS include anovulation and polycystic ovary, systemic CLGI, and the accumulation of excess adipose tissue, including visceral fat. The results of the latest studies on the therapeutic potential of berberine in PCOS are promising, suggesting that it can disrupt the self-perpetuating cluster of disorders.

The main barrier to the therapeutic use of berberine is its extremely low oral bioavailability caused by extensive intestinal first-pass elimination and predominant hepatic metabolism of this compound. Paradoxically, the notoriously low bioavailability of berberine makes it highly effective as a gut-targeted therapy, directly allowing it to act on the gastrointestinal microbiome for the treatment of gut dysbiosis. Because PCOS often involves reduced microbial diversity, systemic CLGI, and altered hormone metabolism, optimizing the gut microbiome is becoming an important complementary strategy to standard medical care. In addition to gut-targeted therapies, increasingly effective methods are being developed to ensure optimal (therapeutic) blood levels of berberine by enhancing its intestinal absorption and inhibiting its metabolism.

The variable efficacy of berberine in treating PCOS may stem from the high heterogeneity of this condition, with the greatest efficacy expected in classic forms of PCOS characterized by a high degree of metabolic dysfunction driven by IR. Moreover, berberine is regulated as a dietary supplement, meaning that it bypasses the rigorous FDA approval process required for prescription drugs. Consequently, there is no standardized dosing, limited regulatory oversight, and no official guarantee regarding the potency or purity of commercial supplements.

Berberine and metformin share significant biological overlap, primarily because both act as AMPK activators. Because they both regulate blood sugar levels, reduce lipid levels, and promote weight management, berberine is frequently referred to as “nature’s metformin”. However, the use of metformin as an FDA-approved prescription drug is backed by thousands of large-scale, long-term clinical trials, whereas the body of research on berberine—when used as a supplement—consists largely of shorter, smaller studies. Therefore, replacing metformin with berberine in the treatment of PCOS or other metabolic diseases that develop on the basis of IR and hyperinsulinemia is not currently clinically feasible.

## Figures and Tables

**Table 1:** Beneficial effects of berberine in the treatment of PCOS-related metabolic/hormonal disorders.

Observed Anti PCOS-Effect of Berberine	Mechanism of Action	Expected Therapeutic Effects	References	Types of Research Conducted/Cited
Reversing insulin resistance [IR]	Activation of the AMP-activated protein kinase (AMPK) pathway.	Lowering fasting blood glucose, decreased serum insulin concentration, reducing risk of developing type 2 diabetes (T2D)	Liu et al., 2025 [150]; Turner et al., 2008 [241]; Jin et al., 2024 [242]; Kamalakannan et al., 2026 [243]; Zhang et al., 2021 [244]	Animal models (mouse, rat); in vitro studies (cell culture, isolated muscle mitochondria)
Reducing hyperandrogenism	Multi-axis regulatory network including increase in SHBG production in the liver by improving insulin sensitivity, direct inhibition of ovarian androgen synthesis in theca cells through downregulation of steroidogenic enzymes, suppression of enzymes converting weak hormone precursors into dihydrotestosterone (DHT), downregulation of the steroidogenic acute regulatory (StAR) protein, stimulation of aromatase by increase in the gene CYP19a1 expression, and suppression of androgen receptor expression. Indirect inhibition of adrenal androgen production via the hypothalamic–pituitary–adrenal (HPA) axis by limiting hyperinsulinemia.	Clearance of physical symptoms (acne, hirsutism, and alopecia), restoration of normal ovarian morphology (disappearance of cysts), return of regular ovulatory menstrual cycles, reducing visceral fat accumulation and inflammation in adipose tissue, and prevention of long-term metabolic and cardiovascular complications.	Jurgiel et al., 2024 [239]; Di Perro et al., 2023 [245]; Wu et al., 2025 [246]; Xie et al., 2019 [247]	Randomized controlled trial; single-arm pilot study; in vitro studies (cell culture); comparative clinical study; meta-analysis of randomized controlled trials
Restoring ovulation and fertility	Reducing IR through activation of the AMPK pathway with subsequent limitation of ovarian and adrenal hyperandrogenism and restoration of the normal LH/FSH ratio and pulsatile release of gonadotropin-releasing hormone (GnRH).	The ovaries regain normal morphology (cysts disappear), cycles become regular and ovulatory, reduced oxidative stress and inflammation within the follicular microenvironment protects ovarian granulosa cells. Both oocyte quality and endometrial receptivity improve.	Jurgiel et al., 2024 [239]; Ionescu et al., 2023 [240]; Wang et al., 2021 [248]; Gao et al., 2026 [249]; Liu et al., 2024 [250]	Randomized controlled trial; animal model (mouse, rat); single-arm pilot study; retrospective case–control study
Counteracting chronic low-grade inflammation (CLGI)	Suppression of key pro-inflammatory signaling pathways, including those associated with nuclear factor kappa-light-chain-enhancer of activated B cells (NF-κB) and mitogen-activated protein kinase (MAPK), as well as activating the AMPK pathway.	Decreased serum levels of CLGI biomarkers such as high sensitivity C-reactive protein (hs-CRP), TNF-α, IL-1β, and IL-6. Reducing tissue expression of pain-inducing enzymes such as cyclooxygenase-2 (COX-2) and inducible nitric oxide synthase (iNOS). Shift in macrophage phenotype from the pro-inflammatory M1 to the anti-inflammatory M2, promoting tissue repair.	Sun et al., 2024 [83]; Lu Y et al., 2022 [251]; Shin et al., 2016 [252]; Sun et al., 2018 [253]	Animal model (mouse); meta-analysis of randomized controlled trials; in vitro studies (cell culture, isolated muscle mitochondria)
Improvement in body composition and weight management	AMPK activation leads to decreased lipogenesis (turns off enzymes required to synthesize new fat) and increases fat burning by triggering the movement of fatty acids into the mitochondria for oxidation.	Reduction in visceral fat mass and lowering waist-to-hip ratio, even in the absence of overall weight loss. Lowering the body mass index (BMI).	Amini et al., 2020 [254]; Rondanelli et al., 2021 [255]; Liu et al., 2020 [256]; Asbaghi et al., 2020 [257]; Rong et al., 2022 [258]	Meta-analysis of randomized controlled trials; one-group pretest/post-test study; in vitro study (cell culture); meta-analysis of randomized controlled trials; animal model (mouse)
Normalization of free fatty acid (FFA) levels and cholesterol profiles	Increased burning of FFAs after AMPK activation preventing fat storage. Inhibition of specific receptors, including peroxisome proliferator-activated receptor gamma (PPARγ) and fatty acid translocase/cluster of differentiation 36 (FAT/CD36), reducing the cellular uptake of FFAs in muscle tissue, thereby reversing IR. Upregulation of low-density lipoprotein (LDL) receptors (LDLRs) increases clearance of “bad” cholesterol from the bloodstream.	A significant improvement in insulin sensitivity and a reduction in systemic CLGI. Shifts in blood lipid markers with reduced total cholesterol (TC), LDL-cholesterol (LDL-C), and decreased triglycerides (TGs). Improvement in the course of metabolic dysfunction-associated steatotic liver disease (MASLD) with increased production of sex hormone-binding globulin (SHBG).	Sun et al., 2017 [259]; Pirillo & Catapano, 2015 [260]; Li et al., 2025 [261]; Lei et al., 2026 [262]	In vitro study (cell cultures); animal models (hamster, mouse, rat); meta-analysis of randomized controlled trials
Optimization of cardiovascular health	Activation of AMPK signaling alleviates endoplasmic reticulum (ER) stress and normalizes reactive oxygen species (ROS) accumulation in vessel wall; upregulation of endothelial nitric oxide synthase (eNOS) stimulates nitric oxide (NO) release; downregulation of NOX4 (NADPH oxidase 4) enzymatic protein reduces production of oxidative stress mediators (e.g., hydrogen peroxide); inhibition of endothelial microparticles (EMPs) protects from vascular damage; suppressing inflammation by inhibiting the Akt (protein kinase B)/interferon regulatory factor 3 (IRF3) pathway; normalization of FFA levels and cholesterol profiles (see the table row above).	Increased insulin sensitivity (reversal of IR); lowering cardiovascular risk resulting from reduced toxic lipid exposure with limited lipid peroxidation-driven cell death (ferroptosis) and cellular injury in vascular endothelial cells; antiplatelet aggregation and antiatherosclerotic effects by improving vascular endothelial function with limited vascular inflammation and inhibition of the formation of atherosclerotic plaques; blood pressure regulation by dilation of blood vessels.	Zhao et al., 2026 [102]; Liu et al., 2015 [131]; Tan et al., 2020 [263]; Zhao et al., 2021 [264]; Zamani et al., 2022 [265]	Randomized controlled trial; cohort studies, Mendelian randomization; animal models (mouse, rat); human dose response meta-analysis
Optimization of gut health	Changing the composition of gut microbiota by promoting beneficial, short-chain fatty acid (SCFA)-producing bacteria while inhibiting pathogenic strains. These SCFAs (e.g., butyrate, acetate, propionate) activate the AMPK signaling pathway, which decreases blood glucose, lowers lipids, improves insulin sensitivity, and lowers systemic endotoxins (LPS) in the blood. This downregulates the toll-like receptor (TLR4)/NF-κB signaling pathways, significantly reducing pro-inflammatory cytokines and protecting the intestinal mucosal barrier (upregulating “tight junction” proteins). Berberine’s microbiota-driven metabolites influence host immunity and cell survival by suppressing the phosphatidylinositol 3-kinase (PI3K)/protein kinase B (Akt) pathway and modulating mammalian target of rapamycin (mTOR) to maintain cellular autophagy. Berberine suppresses the activity of bacterial bile salt hydrolase (BSH), activating the intestinal farnesoid X receptor (FXR) and its co-expressing G protein-coupled bile acid receptor 1 (TGR5), which has antiinflammatory effects and results in the stimulation of glucagon-like peptide-1 (GLP-1) secretion with subsequent increase in pancreatic insulin release.	Improved metabolic health as evidenced by reduced IR, lower cholesterol and better lipid profiles; improvement of hepatic metabolism resulting in increased production of SHBG; anti-inflammatory effects with promotion of the growth of butyrate-producing microbes, which helps restore the intestinal barrier integrity and reduces metabolic endotoxemia; decreased serum levels of CLGI biomarkers and macrophage infiltration specifically within visceral adipose tissue.	He et al., 2022 [266]; Wang et al., 2022 [267]; Zhang et al., 2021 [268]; Cheng et al., 2022 [269]	Meta-analysis of randomized controlled trials; randomized controlled trial; animal studies (hamster, mouse, rat); bacteriological in vitro studies

## Data Availability

No new data were created or analyzed in this study. Data sharing is not applicable to this article.

## Abbreviations

The following abbreviations are used in this manuscript:

A4	androstenedione
ACAT2	acetyl-CoA-cholesterol acyltransferase 2
ACE	angiotensin-converting enzyme
ACTH	adrenocorticotropic hormone
AJs	adherens junctions
Akt	protein kinase B
AMPK	5′ adenosine monophosphate (AMP)-activated protein kinase
APCs	antigen-presenting cells
ARC	arcuate nucleus
ASC	apoptosis-associated speck-like protein containing a caspase recruitment domain
ATF6	activating transcription factor 6
ATR1	angiotensin receptor 1
BAT	brown adipose tissue
BMI	body mass index
BSH	bile salt hydrolase
CHOP	C/EBP homologous protein, also known as DDIT3 or GAGG153
CLGI	chronic low-grade inflammation
COX-2	cyclooxygenase 2
CVDs	cardiovascular diseases
CYP	cytochrome P450
DCs	dendritic cells
DDIT3	DNA damage-inducible transcript 3, also known as CHOP or GAGG153
DES	deep eutectic solvent (extraction)
DHEA	dehydroepiandrosterone
DHEAS	dehydroepiandrosterone sulfate
DHT	dihydrotestosterone
EMPs	endothelial microparticles
EMT	epithelial–mesenchymal transition
eNOS	endothelial nitric oxide synthase
ER	endoplasmic reticulum
ERK1/2	extracellular signal-regulated kinases 1 and 2
ESR	erythrocyte sedimentation rate
FAT/CD36	fatty acid translocase/cluster of differentiation 36
FDA	Food and Drug Administration
FFAs	free fatty acids
FXR	farnesoid X receptor
GABA	gamma-aminobutyric acid
GAGG153	growth arrest and DNA damage-inducible protein 153, also known as CHOP or DDIT3
GLP-1	glucagon-like peptide-1
GLUT4	glucose transporter type 4
GnRH	gonadotropin-releasing hormone
HbA1c	glycosylated hemoglobin
HDL	high-density lipoprotein
HDL-C	high-density lipoprotein cholesterol
HFD	high-fat diet
HOMA-IR	Homeostatic Model Assessment of Insulin Resistance
HPA	Hypothalamic–pituitary–adrenal (axis)
HPG	Hypothalamic–pituitary–gonadal (axis)
hs-CRP	high-sensitivity C-reactive protein
IκBα	inhibitory protein kappa B alpha
IFN-γ	interferon gamma
IHD	ischemic heart disease
IKK	inhibitory protein kappa B alpha (IκBα) kinase
IL-1β, IL-6, IL-17A, IL-17F, IL-21, IL-22	interleukins: -1β, -6, -17A, -17F, -21 and -22, respectively
iNOS	inducible nitric oxide synthase
IR	insulin resistance
IRE1	inositol-requiring enzyme 1
IRF8	interferon regulatory factor 8
IUPAC	International Union of Pure and Applied Chemistry
IVF	In Vitro fertilization
JAK	Janus kinase
JNK	c-Jun NH2-terminal kinases
KNDy neurons	kisspeptin, neurokinin B, dynorphin neurons
LDH	lactate dehydrogenase
LDL-C	low-density lipoprotein cholesterol
LDLRs	low-density lipoprotein (LDL) receptors
LH	luteinizing hormone
LPS	lipopolysaccharide
MAE	microwave-assisted extraction
MASLD	metabolic dysfunction-associated steatotic liver disease
MDA	malondialdehyde
MIRI	myocardial ischemia–reperfusion injury
MMP-2, MMP-9	matrix metalloproteinases -2 and -9
MMPs	matrix metalloproteinases
MAPK	mitogen-activated protein kinase
mTOR	mechanistic target of rapamycin
MYD88	myeloid differentiation primary response 88
NAFLD	non-alcoholic fatty liver disease
NF-κB	nuclear factor kappa-light-chain-enhancer of activated B cells
NO	nitric oxide
NOD	nucleotide-binding oligomerization domain
OGTT	oral glucose tolerance test
PCOS	polycystic ovary syndrome
PCSK9	proprotein convertase subtilisin/kexin type 9
PERK	protein kinase R-like ER kinase
P-gp	P-glycoprotein
PI3K	phosphatidylinositol 3-kinase
PPARγ	peroxisome proliferator-activated receptor gamma
PV	plasma viscosity
RAA	renin–angiotensin–aldosterone
RhoA	Ras homolog family member A
ROCK	Ras homolog (Rho)-associated protein kinase
ROS	reactive oxygen species
RVLM	rostral ventrolateral medulla
SCFAs	short-chain fatty acids
SERCA	sarcoplasmic/endoplasmic Ca^2+^–ATPase
SFE	supercritical fluid extraction
SHBP	sex hormone-binding globulin
SIRT1	sirtuin 1
StAR protein	steroidogenic acute regulatory protein
STAT	signal transducers and activators of transcription
STAT1	signal transducer and activator of transcription 1
STAT3	signal transducer and activator of transcription 3
T2D	type 2 diabetes
TC	total cholesterol
TCM	traditional Chinese medicine
TGR5	G protein-coupled bile acid receptor 1
TGs	triglycerides
Th1	T helper type 1 cell, a lineage of CD4+ effector T-cell (lymphocyte)
Th17	T helper type 17 cell, a lineage of CD4+ effector T-cell (lymphocyte)
TJs	tight junctions
TLR4	toll-like receptor 4
TLRs	toll-like receptors
TNF-α	tumor necrosis factor alpha
Tregs	regulatory T-cells
UPE	ultrahigh pressure extraction
UPR	unfolded protein response
USE	ultrasonic-assisted extraction
VEGF	vascular endothelial growth factor
VSMCs	vascular smooth muscle cells
WBC	white blood cell count
